# Cysteine Attenuates Intestinal Inflammation by Regulating the Gut Microbiota and TLR4-JNK/MAPK-NF-κB Pathway in Piglets

**DOI:** 10.3390/ijms262411991

**Published:** 2025-12-12

**Authors:** Rui Liu, Pengxiang Qin, Zihao Liu, Wenjing Liu, Shuzhen Jiang, Xuejun Yuan, Weiren Yang, Caiyun Huang, Ning Jiao

**Affiliations:** 1 Key Laboratory of Efficient Utilization of Non-Grain Feed Resources (Co-Construction by Ministry and Province), College of Animal Science and Technology, Ministry of Agriculture and Rural Affairs, Shandong Agricultural University, Tai’an 271017, China; 2 College of Life Sciences, Shandong Agricultural University, Tai’an 271018, China; 3 College of Animal Science and Technology, Fujian Agriculture and Forestry University, Fuzhou 350002, China

**Keywords:** intestine, cysteine, inflammation, gut microbiota

## Abstract

As a nutritionally important amino acid, cysteine (Cys) could attenuate oxidative damage on growth performance and intestinal barrier function in piglets. However, the mechanism of Cys in attenuating intestinal injury remains unclear. The aim of this study was to investigate the mechanism of Cys in defending against intestinal inflammation in piglets. A total of twenty-four piglets were divided into four groups and fed a diet with or without 0.1% BPA or Cys for a 28 d feeding trial. The results showed that Cys supplementation reinstated the jejunal barrier by increasing cell proliferation and the goblet cell number, and decreased cell apoptosis upon BPA exposure. Cys supplementation also decreased serum and jejunal pro-inflammatory cytokine and immunoglobulin levels in BPA-challenged piglets. Furthermore, Cys mitigated inflammation by normalizing the activation of the toll-like receptor 4 (TLR4)-JNK/MAPK-nuclear factor-kappa B (NF-κB) pathway caused by BPA. Additionally, dietary Cys supplementation restored the levels of butyrate, valerate and isovalerate in cecum contents that were decreased by BPA exposure. Meanwhile, Cys supplementation normalized the abundances of *Prevotellaceae* and *Romboutsia* upon BPA exposure. In conclusion, Cys is critical to nutrition through attenuating intestinal inflammation by regulating gut microbial balance and suppressing the TLR4-JNK/MAPK-NF-κB pathway.

## 1. Introduction

The intestine is the primary organ for nutrient digestion and absorption, and its epithelial barrier serves to defend against pathogenic bacteria [1]. Accumulating evidence indicates that intestinal function is influenced by a variety of factors, such as toxins, infections and oxidative stress, which can lead to intestinal mucosa damage and dysfunction, such as with inflammatory bowel disease (IBD) [2,3]. In addition, as the biological intestinal barrier, the gut microbiota plays a crucial role in maintaining intestinal health by regulating nutrient metabolism, intestinal epithelial integrity, and the immune system [4]. In addition, studies have shown that the gut microbiota participates in the immune response by alleviating intestinal inflammation [5,6]. Bisphenol A (BPA), which is commonly present in daily commodities, can induce inflammatory responses, reducing the species diversity of the gut microbiota in piglets [6,7]. This indicates that it is necessary to regulate the gut microbiota and immune response in order to maintain intestinal health.

Cysteine (Cys), as a type of amino acid and carrier of a thiol group, plays a key role in promoting the synthesis of glutathione (GSH) [8]. The relatively low bioavailability of Cys is considered to be consumed by 25% of the first-pass metabolism of piglets and mainly participates in the synthesis of intestinal mucosal epithelial proteins [9]. Cys could alleviate oxidative stress in inflammatory bowel disease [10]. In particular, Cys has beneficial effects on growth performance, inflammatory response and intestinal mucosal integrity, and exerts a role independent of GSH [10,11]. Previous studies have shown that Cys can effectively alleviate BPA-induced oxidative damage in piglets by increasing the average daily gain, improving antioxidant capacity and jejunal morphology [12]. However, the mechanism of Cys in attenuating intestinal injury is not fully understood.

The aim of this study was to investigate the potential mechanism of Cys in attenuating BPA-induced inflammation and regulating intestinal microbial composition in piglets. The results of this study could contribute to a better understanding of the potential mechanisms of Cys in alleviating intestinal inflammation and provide a foundation for Cys application in the pig industry.

## 2. Results

### 2.1. Jejunal Mucosal Cell Renewal

**Figure 1:**
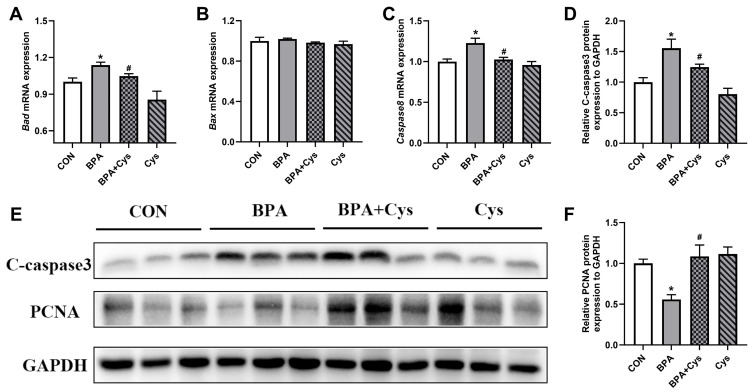
Cysteine alleviated BPA-induced disorder in jejunal cell renewal. (**A**–**C**) mRNA expression of *Bcl-2 associated death promoter* (*Bad*), *Bcl-2 associated X* (*Bax*), and *Caspase 8*, respectively. (**E**) Protein expression of C-caspase 3 and proliferating cell nuclear antigen (PCNA). (**D**,**F**) Statistical analysis of Western blot using Fusion software (16.0.9.0). Results are expressed as means ± SEMs, *n* = 6 or 3. * *p* < 0.05 as compared to the CON group. ^#^ *p* < 0.05 as compared to the BPA group.

To investigate the effects of Cys on alleviating cell renewal upon BPA exposure, we evaluated cell proliferation and apoptosis in the jejunal mucosa. Compared with the CON group, BPA exposure significantly increased the mRNA expression of *Bad* and *Caspase 8* (Figure 1A–C) (*p* < 0.05). However, Cys supplementation significantly decreased those expressions upon BPA exposure (*p* < 0.05). Moreover, BPA increased the protein abundance of C-caspase 3, while decreasing PCNA abundance (Figure 1D–F) (*p* < 0.05). However, Cys markedly restored the expression of C-caspase 3 and PCNA upon BPA exposure (*p* < 0.05). There were no significant differences in the mRNA expression of *Bad*, *Bax* and *caspase 8* and the protein abundance of C-caspase 3 and PCNA between the control and BPA + Cys groups.

### 2.2. Jejunal Goblet Cell Distribution

**Figure 2:**
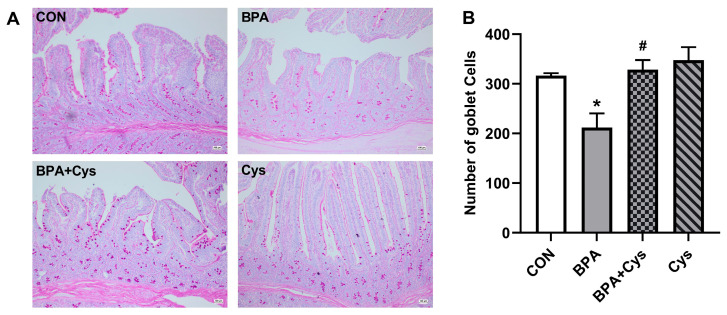
Cysteine alleviated BPA-induced piglet jejunal injury. (**A**) Goblet cells were stained with PAS. (**B**) Statistical analysis of goblet cell number in jejunum. Results are presented as means ± SEMs, *n* = 3. Scale bars indicate 100 μm. * *p* < 0.05 as compared to the CON group. ^#^ *p* < 0.05 as compared to the BPA group.

To evaluate the potential intestinal morphology alternations, PAS staining was used to detect the effects of BPA and Cys on the number of goblet cells. As shown in Figure 2, the mucin layer became thinner, and goblet cells were sparsely distributed and less evident in the BPA group compared to the CON group. However, dietary Cys supplementation restored the mucin layer. In addition, goblet cells were relatively densely distributed and more abundant in the BPA + Cys group compared to the BPA group.

### 2.3. Serum Inflammatory Factors and Immunoglobulin Content

**Figure 3:**
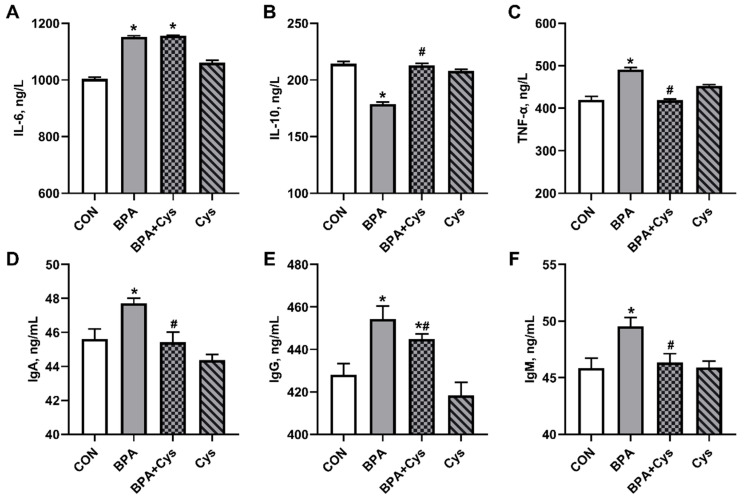
Effects of dietary cysteine supplementation on serum inflammatory factor and immunoglobulin contents in BPA-challenged piglets: (**A**) interleukin-6; (**B**) interleukin-10; (**C**) tumor necrosis factor-α; (**D**) immunoglobulin A; (**E**) immunoglobulin G; and (**F**) immunoglobulin M. Results are expressed as means ± SEMs, *n* = 6. * *p* < 0.05 as compared to the CON group. ^#^ *p* < 0.05 as compared to the BPA group.

Compared with the CON group, BPA challenge significantly increased the serum levels of IL-6, TNF-α, IgA, IgG and IgM (Figure 3A,C,D–F) (*p* < 0.05), while decreasing the levels of IL-10 in the serum (Figure 3B) (*p* < 0.05). Under the condition of BPA challenge, cysteine supplementation significantly reduced the levels of TNF-α, IgA, IgG and IgM in the serum (*p* < 0.05) and increased the content of IL-10 in serum upon BPA exposure (*p* < 0.05). However, cysteine supplementation did not restore the concentration of IL-6 induced by BPA (*p* < 0.05). IgG content in the BPA + Cys group was higher than that in the control group (*p* < 0.05).

### 2.4. Jejunal Mucosa Inflammatory Factor and Immunoglobulin Contents

**Figure 4:**
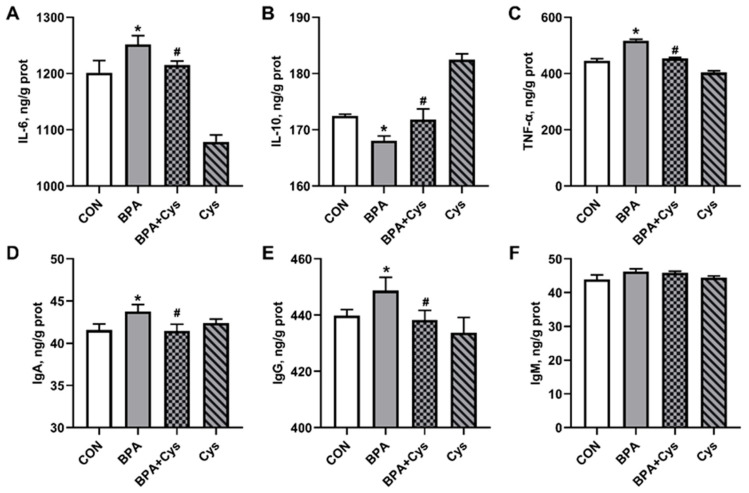
Effects of dietary cysteine supplementation on jejunal mucosa inflammatory factors and immunoglobulin contents in BPA-challenged piglets: (**A**) interleukin-6; (**B**) interleukin-10; (**C**) tumor necrosis factor-α; (**D**) immunoglobulin A; (**E**) immunoglobulin G; and (**F**) immunoglobulin M. Results are expressed as means ± SEMs, *n* = 6. * *p* < 0.05 as compared to the CON group. ^#^ *p* < 0.05 as compared to the BPA group.

According to Figure 4, the intestinal concentrations of IL-6, TNF-α, IgA and IgG in the BPA group were significantly higher than those in the CON group (Figure 4A,C–E) (*p* < 0.05), while the concentration of IL-10 was lower in the jejunal mucosa (Figure 4B) (*p* < 0.05). However, cysteine supplementation reversed the contents of IL-6, IL-10, TNF-α, IgA and IgG upon BPA exposure (*p* < 0.05).

### 2.5. Jejunal Inflammation-Related Index Expression

**Figure 5:**
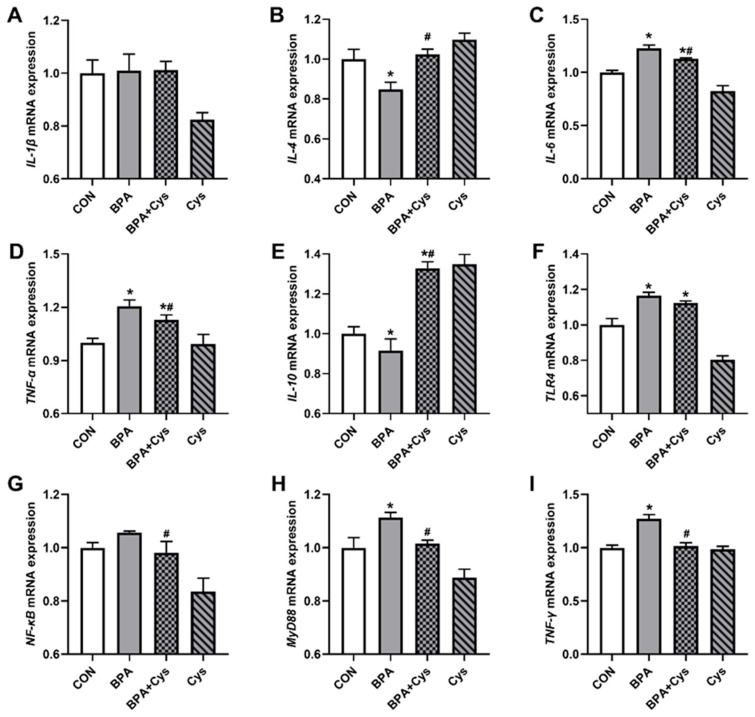
Cysteine alleviated the BPA-induced mRNA expression of inflammatory-relative indexes in the jejunal mucosa: (**A**) *interleukin-1β* (*IL-1β*); (**B**) *interleukin-4* (*IL-4*); (**C**) *interleukin-6* (*IL-6*); (**D**) *tumor necrosis factor-α* (*TNF-α*); (**E**) *interleukin-10* (*IL-10*); (**F**) *toll-like receptor 4* (*TLR4*); (**G**) *nuclear factor kappa-B* (*NF-κB*); (**H**) *myeloid differentiation factor 88* (*MyD88*); and (**I**) *interferon-γ* (*IFN-γ*). Results are expressed as means ± SEMs, *n* = 6. * *p* < 0.05 as compared to the CON group. ^#^ *p* < 0.05 as compared to the BPA group.

The effects of Cys on BPA-induced inflammation-relative indexes’ mRNA expression in piglets’ jejunal mucosa are shown in Figure 5. Compared to the CON group, the mRNA expression of *IL-6*, *TNF-α*, *TLR4*, *MyD88* and *TNF-γ* in the BPA group was significantly increased and that of *IL-10* and *IL-4* was significantly decreased (*p* < 0.05). However, the mRNA expressions of *IL-6*, *TNF-α*, *TLR4*, *NF-κB*, *MyD88* and *TNF-γ* in the BPA + Cys group were obviously lower and that of *IL-10* and *IL-4* was obviously higher than in the BPA group (*p* < 0.05). In addition, Cys supplementation decreased the mRNA expression of NF-κB upon BPA exposure (*p* < 0.05).

### 2.6. Cysteine Attenuated the Induction of NF-κB, TLR4 and p-p38 in BPA-Challenged Piglet Jejunum

**Figure 6:**
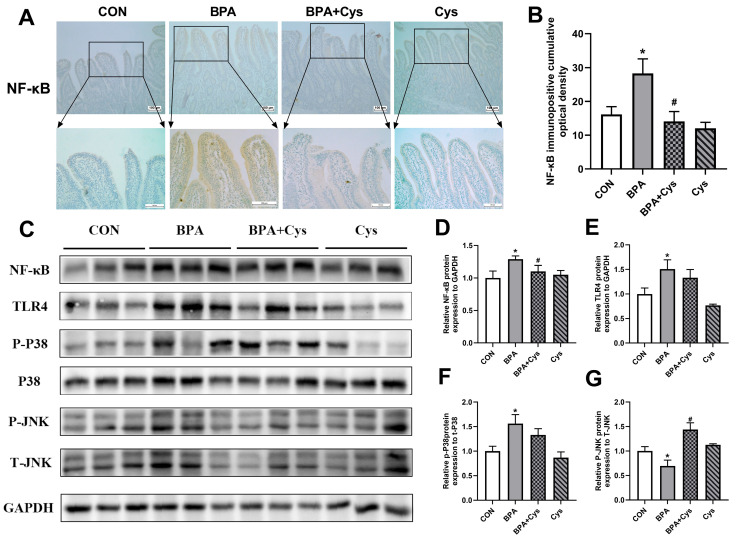
Effects of Cys supplementation on TLR4-NF-κB-MAPK signaling in BPA-challenged piglets. (**A**) Representative immunohistochemistry staining images of NF-κB in jejunum. (**B**) Statistical analysis of (**A**). Scale bars are 100 µm for the above set of figures and 20 µm for the below set of figures, *n* = 6. (**C**) Protein abundance of NF-κB and TLR4, P38 and JNK phosphorylation were measured by Western blot. (**D**–**G**) Statistical analysis of Western blot using Fusion software (16.0.9.0). Results are expressed as means ± SEMs, *n* = 3. * *p* < 0.05 as compared to the CON group. ^#^ *p* < 0.05 as compared to the BPA group.

The immunohistochemical analysis showed that the light brown granular immune-reactive substances of NF-κB were mainly located in the intestinal villous cells and crypt cells of the piglet jejunum (Figure 6A). The NF-κB immune-reactive cells were densely distributed in the intestinal villous and crypt cells of the jejunum in the BPA group, compared with those in the CON group (Figure 6A,B) (*p* < 0.05). However, there were fewer NF-κB immune-reactive cells in the BPA + Cys group than in the BPA group (*p* < 0.05). In addition, BPA induced the activation of NF-κB and TLR4, and the phosphorylation of p38 and JNK (Figure 6C–G) (*p* < 0.05). However, Cys supplementation restored the abundance of NF-κB and phosphorylation of JNK upon BPA exposure.

### 2.7. Concentration of SCFAs in Cecal Content

**Figure 7:**
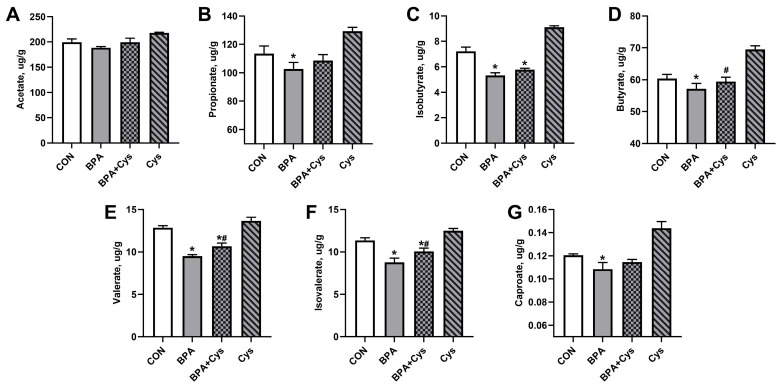
Volatile fatty acid content in the cecal content: (**A**) acetate; (**B**) propionate; (**C**) isobutyrate; (**D**) butyrate; (**E**) valerate; (**F**) isovalerate; and (**G**) caproate. Results are expressed as means ± SEMs, *n* = 6. * *p* < 0.05 as compared to the CON group. ^#^ *p* < 0.05 as compared to the BPA group.

Compared to the CON group, BPA exposure led to a decrease in propionate, isobutyrate, butyrate, valerate, isovalerate and caproate levels in cecal content (Figure 7B–G). The levels of butyrate, valerate and isovalerate increased in the BPA + Cys group compared with the BPA group (*p* < 0.05) (Figure 7D–F). In addition, valerate and isovalerate levels were lower in the BPA + Cys group than in the control group (*p* < 0.05) (Figure 7E,F). There was no significant difference in acetate content between the groups (Figure 7A) (*p* > 0.05).

### 2.8. Diversity of Gut Microbiota

**Figure 8:**
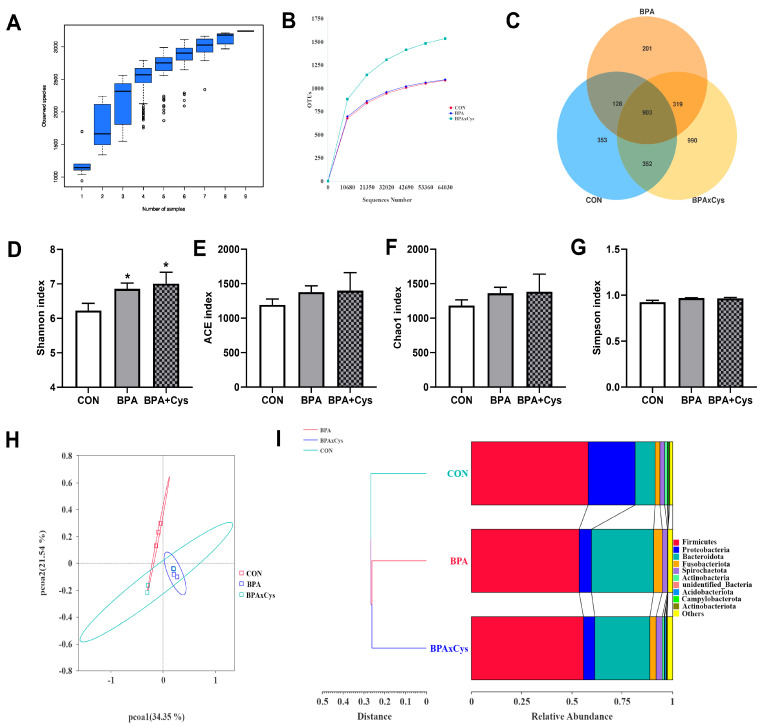
Effects of Cys supplementation on gut microbiota in BPA-challenged piglets. (**A**) The species accumulation curve. (**B**) The rarefaction curve of OTUs. (**C**) Venn diagram. (**D**) Shannon index. (**E**) ACE index. (**F**) Chao 1 index. (**G**) Simpson index. (**H**) Principal coordinate analysis (PCoA). (**I**) Phylogenetic tree using bray_curtis algorithm. Results are expressed as means ± SEMs, *n* = 3. * *p* < 0.05 as compared to the CON group.

As shown in Figure 8A, the species accumulation curves tended to flatten as the number of analyzed sequences increased, indicating that the samples were sufficient for OTU testing to predict their species richness. The rarefaction curve tended to approach the asymptote, indicating that the sequence depth was also sufficient to represent most species richness and bacterial community diversity (Figure 8B). Based on a 97% sequence similarity, a total of 3246 OTUs were identified in this experiment, and the CON, BPA, and BPA + Cys groups contained 353, 201, and 990 unique OTUs, respectively (Figure 8C). Compared with the control group, BPA exposure significantly increased the Shannon index (Figure 8D) (*p* < 0.05). However, there were no significant differences in ACE, Chao1, and Simpson indexes among the three groups (Figure 8E–G) (*p* > 0.05). The PCoA analysis revealed that the BPA samples dispersed far from the CON samples, suggesting a clear separation between the BPA and CON groups (Figure 8H). Moreover, the UPGMA phylogenetic tree showed that the BPA and BPA + Cys groups were close together and clustered in one group, while the CON group was distributed in a separate branch (Figure 8I).

### 2.9. Abundance of Microbiota in the Cecal Content

**Figure 9:**
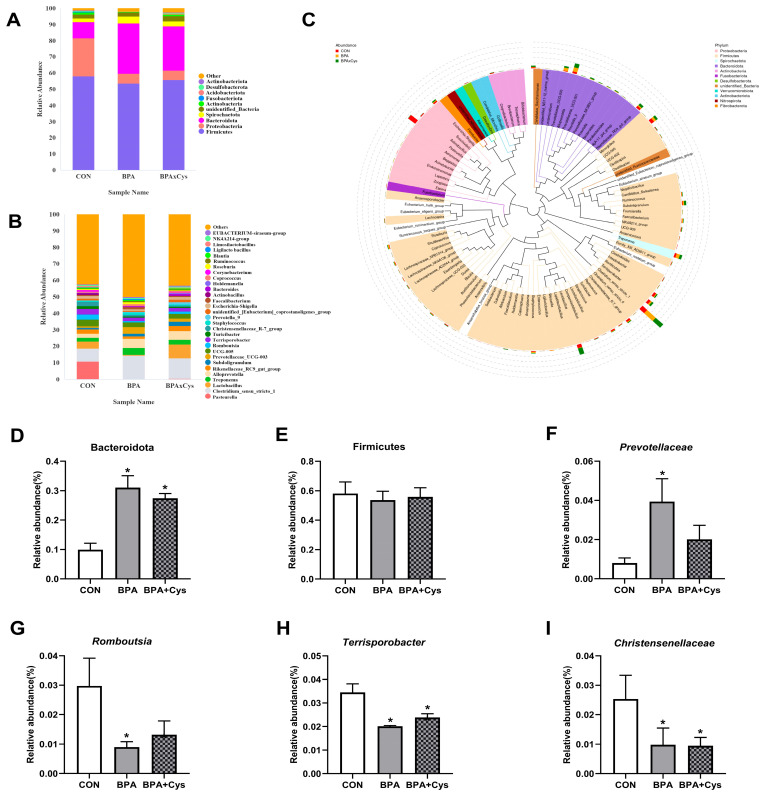
Effects of Cys supplementation on microbiota phylum and genus abundance in BPA-challenged piglets. (**A**) Abundance of gut microbiota at the phylum level (top 10). (**B**) Abundance of gut microbiota at the genus level (top 30). (**C**) Phylogenetic tree constructed based on the sequences of the top 100 genera. Relative abundance of (**D**) Bacteroidota, (**E**) Firmicutes, (**F**) *Prevotellaceae*, (**G**) *Romboutsia*, (**H**) *Terrisporobacter*, and (**I**) *Christensenellaceae*. Results are expressed as means ± SEMs, *n* = 3. * *p* < 0.05 as compared to the CON group.

Figure 9A,C show the abundance of the top 10 phyla in the cecal microbiota. The most abundant phyla identified were Firmicutes and Bacteroidota, accounting for 58% and 23%, respectively. BPA exposure significantly increased the abundance of Bacteroidota (*p* < 0.05), while Cys supplementation did not alter the microbiota abundance at the phylum level compared to the BPA group (Figure 9D–F) (*p* > 0.05).

Figure 9B,C display the abundances of the top 30 species at genus level. The genus-level speciation tree shows that the relative abundance of Firmicutes was made up of *Clostridium*, *Terrisporobacter*, *Romboutsia*, *Lactobacillus*, *Subdoligranulum* and *UCG-005*. Bacteroidota was mainly distributed with *Alloprevotella*, *Prevotellaceae_UCG-003* and Rikenellaceae_RC9. BPA significantly increased the abundance of harmful bacteria *Prevotellaceae*, but decreased that of beneficial bacteria *Romboutsia*, *Terrisporobacter* and *Christensenellaceae* (*p* < 0.05). Cys supplementation restored *Prevotellaceae* and *Romboutsia* abundances to normal levels, whereas the abundances of *Terrisporobacter* and *Christensenellaceae* in the BPA + Cys group were lower than those in the control group (*p* < 0.05).

## 3. Discussion

The consumption of BPA-contaminated food or feed can increase intestinal permeability, exacerbate oxidative stress, cause an inflammatory response and reduce gut microbiota diversity [13,14,15]. Our present study confirmed that Cys could attenuate the inflammatory response induced by BPA by regulating the gut microbiota and TLR4-JNK/MAPK-NF-κB signaling in the intestines of piglets. This provides an effective strategy for maintaining intestinal homeostasis and promoting intestine repair after BPA exposure.

The intestinal epithelial barrier maintains the integrity of the gut by protecting against harmful toxins and microorganisms, while also promoting nutrient digestion and absorption [1,16]. The dissociation and deformation of intestinal morphology is a sign of intestinal epithelium injury [17]. The integrity and normal physiological function of the small intestine mucosa are maintained by the continuous renewal of intestinal epithelial cells [18]. In the current study, we found that BPA exposure increased the mRNA expression of apoptosis genes *Bad* and *Caspase 8*, and increased the protein expression of C-caspase 3, as well as decreasing proliferation-related protein PCNA expression in the jejunal mucosa; these findings are similar to those of a previous study [19]. Additionally, it has been reported that Cys decreased enterocyte apoptosis and increased proliferation [20,21]. Similarly, the present study showed that dietary Cys significantly restored the renewal of intestinal epithelial cells by increasing intestinal epithelial cell proliferation and decreasing cell apoptosis upon BPA exposure. Moreover, a previous study has shown that Cys supplementation could relieve BPA-induced morphology damage in the jejunum [12]. Goblet cells play a key role in maintaining barrier function, as they produce mucins and secrete factors that regulate epithelial renewal and healing, and are closely related to the immune system [22,23]. Decreased numbers of goblet cells could increase the risk of pathogens invading the first line of intestinal defense and lead to diseases such as IBD [24,25]. In the present study, Cys supplementation increased the number of goblet cells in the jejunum of piglets exposed to BPA. This indicated that dietary Cys supplementation can reduce damage to the intestinal mucosa upon BPA exposure.

The inflammatory response is a kind of intestinal injury. In addition, injury in the mucosa leads to the activation of the mucosal immune system and the production of pro-inflammatory cytokines [26]. It has been reported that BPA could induce inflammation by upregulating the key factors of the innate immune system and transcriptional activity [6,19]. In addition, this study has shown that Cys supplementation decreased the concentration of serum TNF-α and jejunal IL-6 and TNF-α, while increasing the serum and jejunal concentration of IL-10 in piglets challenged with BPA. Moreover, Cys supplementation reduced the mRNA expression of inflammation-relative genes *IL-6*, *TNF-α*, *NF-κB*, *MyD88* and *TNF-γ*, while significantly decreasing the expression of anti-inflammation-relative genes *IL-10* and *IL-4* in the jejunal tissue. These findings were consistent with a previous study, which showed that Cys reduced the expression of pro-inflammatory cytokines in DSS-induced porcine colonic injury [18]. Immunoglobulins serve as the primary defense against infections, and play a vital role in immune regulation and immunological tolerance [27]. However, we observed that Cys significantly decreased the levels of IgA and IgG in both the serum and jejunal mucosa following exposure to BPA. This may be due to BPA causing inflammation, which then leads to the abnormal secretion of immunoglobulin in the body, while Cys could restore self-adjustment of the intestinal immune system by decreasing the level of immunoglobulins.

It has been shown that TLR4 could activate downstream mediators such as NF-κB, which increases the production of pro-inflammatory factors such as TNF-α, IL-6 [28]. In addition, MAPK has been associated with stress response and inflammation, and the hyper-phosphorylation of MAPK could activate NF-κB [29]. Previous studies have demonstrated that BPA could lead to inflammatory responses through activating JNK/MAPK and TLR4-NF-κB pathways [30]. In this study, we found that BPA increased the expression of TLT4 and NF-κB and phosphorylation of JNK, which was consistent with the previous study [6,31]. Another study showed that Cys supplementation could attenuate LPS-induced inflammation by modulating the NF-κB signaling pathway [20], which was consistent with the findings of the present study. This indicates that Cys may attenuate BPA-induced inflammation by suppressing the TLR4-p38/MAPK-NF-κB signaling pathway.

The intestinal microbial community is involved in a variety of physiological processes, including nutrient digestion and absorption and immune system development [32,33]. A study has reported that BPA exposure can cause gut microbiota dysbiosis by increasing the abundance of harmful bacteria Proteobacteria, while decreasing that of beneficial bacteria Bacteroides, disrupting the homeostatic balance of the microbial community [6]. In this study, we analyzed the gut flora and found that BPA changed the microbiota community structure. Additionally, Cys supplementation increased the abundance of Bacteroidota upon BPA exposure. Bacteroidota are usually considered to be beneficial bacteria in the gut, and can maintain beneficial metabolite production and immunity [34]. A previous study has also found that *Prevotellaceae* has been implicated in IBD, which may disrupt mucosal barrier function through the production of sulfatases that actively degrade mucus oligosaccharides, thus leading to intestinal inflammation [35,36]. In the present study, BPA increased the abundance of *Prevotellaceae*, which was restored by Cys supplementation. Additionally, the decreased abundance of *Romboutsia* caused by BPA was also restored by Cys supplementation. *Romboutsia* plays a key role in maintaining a healthy state in the host, and damage to the gut drastically reduces its abundance, so it is considered to be the first indicator of an alteration to the mucosa [37,38]. *Terrisporobacter* plays an important role in the in vitro anaerobic fermentation of rice straw and is significantly correlated with acetate and total SCFA concentrations [39,40]. *Christensenellaceae* is involved in the production of butyrate and has been shown to be positively associated with a variety of diseases such as ulcerative colitis and Crohn’s disease [41,42]. We found that BPA decreased the abundances of *Terrisporobacter* and *Christensenellaceae*, and the levels of SCFAs. These results indicate that BPA exposure altered the abundance and diversity of the gut bacterial community, thus increasing the percentage of other harmful bacteria and decreasing the percentage of other beneficial bacteria, which may directly affect SCFA production. A study has also shown that SCFAs can promote the proliferation of beneficial bacteria, which can in turn suppress the inflammatory response through the production of SCFAs [43]. Similarly, research suggested that SCFAs control and reduce microbial infections and are indirectly involved in immunomodulation through suppressing NF-κB [33]. In the present study, Cys increased the levels of butyrate, valerate and isovalerate upon BPA exposure, which indicated that Cys could attenuate intestinal inflammation by regulating the intestinal microbiota and promoting SCFA production.

## 4. Materials and Methods

### 4.1. Animals and Experimental Design

The animal experiment was conducted according to the Chinese Guidelines for Animal Welfare and Experimental Protocol and approved by the Animal Care and Use Committee of Shandong Agricultural University (SDAUA-2021-081). A total of 24 healthy 35 d crossbred castrated piglets (Duroc × Lamdrace × Yorkshire) with an initial body weight of 14.45 ± 0.43 kg were randomly divided into four groups with six replicates per group and one piglet per replicate. The control group (CON) was fed a basal diet. The BPA group (BPA) was fed a basal diet supplemented with 0.1% BPA. The BPA + Cys group (BPA + Cys) was fed a diet with 0.11% Cys and 0.1% BPA. The Cys group (Cys) was fed a diet with 0.11% Cys. The doses of Cys and BPA and the experimental diets used in the study were in accordance with a previous study [12].

### 4.2. Sample Collection

After 12 h of fasting, venous blood was collected and serum was obtained by centrifugating blood at 3000 rpm for 15 min at 4 °C, which was then stored at −20 °C until analysis. After blood sampling, all piglets were humanely sacrificed. The intestine of each piglet was quickly removed, and 2 cm length segments were cut from the middle part of the jejunum, washed with saline solution, and fixed with 4% paraformaldehyde solution for morphological analysis. An additional middle portion of the jejunum was opened longitudinally and flushed gently, and then the jejunal mucosa was gently scraped and stored at −80 °C for further analysis. Moreover, cecal content samples were collected into sterile tubes for further analysis.

### 4.3. Determination of Inflammatory Factors and Immunoglobulin Contents

The levels of interleukin-6 (IL-6), interleukin-10 (IL-10), tumor necrosis factor-α (TNF-α), immunoglobulin A (IgA), immunoglobulin G (IgG) and immunoglobulin M (IgM) were measured in the serum and jejunum using enzyme-linked immunoassay (ELISA) kits (Nanjing Jiancheng Institute of Biological Engineering, Nanjing, China). All procedures were performed according to the manufacturer’s protocols.

### 4.4. Identification and Detection of Goblet Cells

The identification and detection of goblet cells were conducted according to a previous study [6]. Briefly, jejunal tissues were fixed in 4% paraformaldehyde solution for 3 d. Then, paraffin sections were prepared and sliced into 6 µm slices. Goblet cells were identified and detected using a PAS staining kit (Nanjing Jiancheng Institute of Biological Engineering, Nanjing, China) according to the manufacturer’s instructions. Intestinal tissue from six pigs in each treatment group was selected to prepare PAS sections. Due to good biological reproducibility, three pigs were chosen per treatment, with six sections cut from each pig for subsequent observation and measurement. Fifteen glands per slide were chosen and the number of goblet cells identified and detected by a researcher who was blinded to the experimental protocol. An OlympusBX41 microscope equipped with a DP25 digital camera (Olympus, Tokyo, Japan) was used for this procedure. The number of goblet cells was calculated per 100 μm of gland length separated from each gland.

### 4.5. Immunohistochemistry Analysis

The jejunal localization and expression of nuclear factor-kappa B (NF-κB) were determined by immunohistochemistry following the procedure outlined in the previous study [6]. Briefly, the prepared slides were treated with 10% hydrogen peroxide to deactivate endogenous peroxidase and then incubated with 10% fetal bovine serum. Afterward, the slides were incubated with NF-κB primary antibody (1:1000) at 4 °C overnight, followed by treatment with HRP anti-rabbit secondary antibody for 1 h at 37 °C. Finally, the slides were stained using the diaminobenzidine tetrachloride (DAB) kit (DAB kit, TIANGEN PA110, Beijing, China). The immunoreactive substances of NF-κB were observed using a Nikon Elipse 80i microscope (Nikon, Tokyo, Japan). The antibodies are listed in Table 1.

### 4.6. Quantitative Real-Time PCR (qRT-PCR) Analysis

The qRT-PCR analysis was conducted in accordance with the previous study [12]. To be specific, total RNA was extracted from jejunal tissues using an AG RNAex Pro (Accurate Biology, Changsha, China), and then reverse-transcribed to cDNA using a PrimeScript RT Reagent Kit (RR037A, TaKaRa Bio Inc., Shiga, Japan). Finally, qRT-PCR was completed using a SYBR^®^ Green Premix Pro Taq HS qPCR Kit (AG11701, Accurate Biology, Dalian, China), according to the manufacturer’s instructions, on a LightCycler 96 (Roch, Basel, Switzerland) real-time PCR system. The relative mRNA expression of the target genes was analyzed with the 2^−ΔΔCt^ method and normalized to *β-actin*. All gene primers are shown in Table 1.

### 4.7. Western Blot Analysis

The jejunal tissues were lysed by RIPA (P0013B, Beyotime, Shanghai, China) supplemented with the protease and phosphatase inhibitor mixture cocktail (P1050, Beyotime, Shanghai, China) to extract total protein. Then, the protein concentration was measured using a BCA kit (CoWin Biotech, Taizhou, China). Western blot measurements were carried out according to the method from a previous study [31]. Briefly, equal amounts of protein (30 μg) were loaded onto sodium dodecyl sulfate polyacrylamide gels and then blotted onto polyvinylidene difluoride membranes (IPVH00010, 0.45 μm, Merck Co., Inc., Hunterton, NJ, USA). The membranes were incubated with primary antibodies (1:1000 dilution) at 4 °C overnight and then with an HRP-conjugated secondary antibody (1:5000 dilution) for 1 h at 25 °C, followed by detection with Fusion FX7 (VILBER, Collégien, France) and quantification using Fusion software (16.0.9.0). Data are expressed as the protein normalized to GPADH expression, and all Western blot experiments were conducted in triplicate and repeated three times. The antibodies are listed in Table 2.

### 4.8. Short-Chain Fatty Acid Concentration in Cecum Content

The concentration of short-chain fatty acids (SCFAs) in cecal content was measured using the gas chromatograph method, as described by the previous study [6]. Briefly, the cecal content (1.5 g) was diluted in water and centrifuged at 15,000× *g* for 10 min at 4 °C. Then, 1 mL of supernatant was transferred and mixed with 0.2 mL metaphosphoric acid and then centrifuged at 15,000× *g* for 10 min. Next, MS analysis of the targeted metabolite was performed using Waters ACQUITY UPLC I-CLASS (Milford, MA, USA) ultra liquid chromatography, equipped with a Waters UPLC BEH C8 column(Milford, MA, USA). Finally, TargetLynx quantitative software (version 4.2, Milford, MA, USA) was used to calculate the peak area of the targeting data, and the retention time was given an allowable error of 15 s. The cecal content concentration calculations were performed using the standard curve method to obtain quantitative results.

### 4.9. Intestinal Microbial Diversity Analysis

Total microbial DNA was extracted from the cecum content using a commercial kit (Omega Bio-Tek, Inc., Norcross, GA, USA) following the manufacturer’s instructions. After determining the concentration, the DNA was diluted to 1 ng/μL using sterile solution. Subsequently, the V4 hypervariable region of the 16S rDNA gene was amplified using 515f and 806r primers (5′-GTGCCAGCMGCCGCGGTAA-3′ and 5′-GGACTACHVGGGTWTCTAAT-3′, respectively). The purified PCR products were used to construct the sequencing library using the AxyPrep DNA Gel Extraction Kit (Axygen Biosciences, Union City, CA, USA), following the manufacturer’s specifications. Purified amplicons were sequenced on the Illumina MiSeq platform (Illumina, Inc., San Diego, CA, USA) according to the standard protocol (Majorbio Bio-Pharm Technology, Co., Ltd., Shanghai, China). The obtained high-quality sequences were clustered as operational taxonomic units (OTUs) based on 97% similarity [44], and then classified to different taxonomic levels with the SILVA database [45] based on the Mothur algorithm to annotate taxonomic information. Alpha diversity indices (Shannon, Simpson, Chao 1 and ACE indexes) were calculated for each sample, and Bray–Curtis distances were calculated and visualized via principal coordinate analysis (PCoA) and UPGMA phylogenetic tree. The alpha and beta diversity of bacterial communities were determined using a Wilcoxon rank-sum test. Significant differences among the microbial communities were assessed with the analysis of similarity (ANOSIM) test.

### 4.10. Statistical Analysis

We performed statistical analyses on the three groups: CON, BPA, and Cys + BPA. All the data were statistically analyzed with one-way ANOVA using SAS (version 9.4, SAS institute, Cary, NC, USA), followed by the Student–Newman–Keuls test. The results are expressed as means ± SEMs. Differences were considered significant at a *p* value < 0.05. The figures were drawn using GraphPad Prism (version 8, La Jolla, CA, USA). * *p* < 0.05 as compared to the CON group; ^#^ *p* < 0.05 as compared to the BPA group.

## 5. Conclusions

In conclusion, the present study revealed that Cys supplementation could attenuate intestinal inflammation by balancing the gut microbiota and suppressing the TLR4-JNK/MAPK-NF-κB signaling pathway.

## Figures and Tables

**Table 1:** Primer sequences for quantitative real-time PCR.

Gene	Primer Sequence (5′ to 3′)	Accession No.	Product Size (bp)
*IL-1β*	F: TTGTCTGTGATGCCAACGTG	XM_021085847.1	108
	R: TGAGGAGGTGGAGAGCCTTC		
*IL-4*	F: GCTTCGGCACATCTACAGACACC	NM_214123.1	110
	R: TCTTGGCTTCATGCACAGAACAGG		
*IL-6*	F: ACCGGTCTTGTGGAGTTTCA	NM_001252429.1	170
	R: GCATTTGTGGTGGGGTTAGG		
*TNF-α*	F: CCAATGGCAGAGTGGGTATG	JF831365.1	116
	R: TGAAGAGGACCTGGGAGTAG		
*IL-10*	F: AACCACAAGTCCGACTCAACGAAG	NM_214041.1	81
	R: GCCAGGAAGATCAGGCAATAGAGC		
*TLR4*	F: CTCCAGCTTTCCAGAACTGC	NM_001113039.2	192
	R: AGGTTTGTCTCAACGGCAAC		
*NF-κB*	F: CTCGCACAAGGAGACATGAA	NM_001048232.1	147
	R: TGAAGAGGACCTGGGAGTAG		
*MyD88*	F: ATTGAAAAGAGGTGCCGTCG	NM_001099923.1	188
	R: CAGACAGTGATGAACCGCAG		
*IFN-γ*	F:AGCTTTGCGTGACTTTGTGT	NM_213948.1	247
	R:ATGCTCCTTTGAATGGCCTG		
*β-Actin*	F: CCACGAAACTACCTTCAACTC	NM_001170517.2	131
	R: TGATCTCCTTCTGCATCCTGT		

**Table 2:** Information regarding antibodies for immunohistochemistry and Western blotting.

Antibodies	Catalog No.	Source	Dilution	Company
PCNA	bs-2006R	Rabbit	1:1000	Bioss, Beijing, China
Cleaved Caspase-3 (Asp175)	#9661	Rabbit	1:2000	Cell Signaling Technology, Danvers, MA, USA
SAPK/JNK	#9252	Rabbit	1:2000	Cell Signaling Technology, Danvers, MA, USA
Phospho-SAPK/JNK (Thr183/Tyr185) (81E11)	#4668	Rabbit	1:2000	Cell Signaling Technology, Danvers, MA, USA
NF-κB p65 (D14E12)	#8242	Rabbit	1:2000	Cell Signaling Technology, Danvers, MA, USA
Toll-like Receptor 4 (D8L5W)	#14358	Rabbit	1:2000	Cell Signaling Technology, Danvers, MA, USA
p38/MAPK	#8690	Rabbit	1:2000	Cell Signaling Technology, Danvers, MA, USA
Phospho-p38/MAPK (Thr180/Tyr182)	#4511	Rabbit	1:2000	Cell Signaling Technology, Danvers, MA, USA
GAPDH	HX1832	Rabbit	1:5000	Huaxingbio, Beijing, China
Anti-rabbit IgG (H+L)	A0208		1:3000	Beyotime, Shanghai, China

## Data Availability

The original contributions presented in this study are included in the article. Further inquiries can be directed to the corresponding author.

## Abbreviations

The following abbreviations are used in this manuscript:

Bad	B-Cell Lymphoma-2 associated death promoter
Bax	B-Cell Lymphoma-2 associated X
BPA	Bisphenol A
Cys	Cysteine
GSH	Glutathione
IFN-γ	Interferon-γ
IgA	Immunoglobulin A
IgG	Immunoglobulin G
IgM	Immunoglobulin M
IL-10	Interleukin-10
IL-1β	Interleukin-1β
IL-4	Interleukin-4
IL-6	Interleukin-6
MyD88	Myeloid differentiation factor 88
NF-κB	Nuclear factor kappa-B
PCNA	Proliferating cell nuclear antigen
SCFAs	Short-chain fatty acid
TLR4	Toll-like receptor 4
TNF-α	Tumor necrosis factor-α
